# Management of tendinopathies among south Indians using collagen II peptide, glucosamine and vitamin C

**DOI:** 10.6026/97320630018558

**Published:** 2022-06-30

**Authors:** Madarapu Balaji, Kadiri Venkata Ranganath, G Pugazhendhi, Vinyas Mayasa, K Barathiraja, R Manimaran, Manoj Prathap Chandran

**Affiliations:** 1 Department of Orthopaedics, Maheshwara Medical College and Hospital, Chitkul, Isnapur, Patancheruvu 502307, Telangana, India; 2 Department of Orthopaedics, Maheshwara Medical College and Hospital, Chitkul, Isnapur, Patancheruvu 502307, Telangana, India; 3 Department of Orthopaedics, Sree Balaji Medical College and Hospital, Chromepet, Chennai 600044, India; 4 Department of Pharmacology, MNR College of Pharmacy, Sangareddy, Telangana, India; 5 Department of General Surgery, Sree Balaji Dental College and Hospital, Pallikaranai, Chennai - 600100, India; 6 Department of General Surgery, Sree Balaji Medical College and Hospital, Chromepet, Chennai - 600044, India; 7 Department of Orthodontics, Sree Balaji Dental College and Hospital, Pallikaranai, Chennai - 600100, India

**Keywords:** Tendinopathy, Tendinitis, collagen II peptide

## Abstract

Tendinopathy is a multi-factorial, broad spectrum of tendon disorder, characterized by activity-related chronic tendon pain and local tenderness. The point of this study was to assess the adequacy of a nutritional supplement containing Glucosamine, type II collagen and vitamin C on the clinical and auxiliary advancement of tendinopathies. The prospective study was Hospital based randomized control trail comparing the efficacy of collage 2 peptide, glucosamine and vitamin c with placebo in various tendinopathies. All diagnosed patients willing for the treatment attending Konaseema Institute of Medical Sciences during period of 2017-2019 were selected with regular follow up of 2nd week, 2nd month & 6 month. The statistics and visualizations of various observations made in the entire study which include a total of 80 patients with various tendinopathies. 60 of them were given collagen 2, glucosamine and vitamin c (cases) and 20 were given placebo (controls). At the end of 6 months almost 90% patients relieved completely of pain. The duration of maximum benefit to reach is almost around 24 weeks. These are seen more commonly to affect non-athletes rather than athletes.

## Background:

Persistent tendinopathy may be a common agonizing condition, regularly seen in reaction to abuse [1-5]. Certain ligaments are more prone to damage than others, just like the Achilles ligament, patellar ligament, and supra spinatus tendon. Traditionalist (non-surgical) treatment is as a rule of troublesome and longstanding event, which might be baffling for both the patients and clinicians [6- 10]. Treatment of unremitting tendinopathy has customarily cantered on controlling irritation and torment, in any case, histological thinks about don't bolster an inflammatory handle within the persistent excruciating ligament, and medications such as corticosteroid infusions and NSAIDs cannot be considered to be shown [11-15]. A recent proof-of-concept study showed a dose-dependent improvement of collagen metabolism in ligaments by the oral administration of gelatine 60 min before jumping exercises. This specific time window was based on the fact that peak availability of free and peptide forms of serum Hydroxyproline is respectively 1 and 2h following the oral ingestion of Gelatin [16- 20].

## Materials and methods:

Hospital based randomized control study was approved by the institution Ethics committee of Konaseema Institute of Medical Sciences, Amalapuram were selected during study period of 2017-2019. The randomized control trail is a pilot study, hence 60 cases and 20 controls were selected. A randomization coding system derived from a computer generated randomization table was followed. After a proper clinical diagnosis patients were selected into two groups according to randomization table.

## Inclusion criteria:

[1] Candidates of middle age (30-60 years) with isolated Tendinopathies only

[2] Candidates with accelerated degenerative changes in tendons.

## Exclusion criteria:

[1] Patient with other comorbidities, any associated infection or compartment syndrome of same limb.

[2] Patients, who are having allergy to glucosamine and collagen II peptides

[3] Patients who had received steroid injection within 3 months for Tendinopathy.

[4] Patients previously treated surgically for Tendinopathy.

Patients will be assessed clinically; a thorough history and a complete physical examination will be carried out. The subjective symptoms and the objective signs will be recorded in a standard proforma. This is followed by a routine investigations as well as an MRI scan of that particular joint or tendon for all those patients whomever it is necessary. A pre- medication and post- medication assessment using the pain assessment scale and functional scale for outcome analysis will be done for all the patients. Total 80 patients of both sexes aged between 30 to 60 years were included. 60 Patients were given oral collagen II peptides, glucosamine and vitamin c (ORTHOBOON) sachet daily once for 90 consecutive days. 20 patients were given oral placebo malto dextrinfor 90 consecutive days. Follow-up for a total 6 months, which is divided into intervals at 2nd week, 2nd month and 6th month. Torment is surveyed by most broadly utilized and acknowledged visual simple scale (Figure 1 - see PDF). It comprises of a 10 centimeter line stamped at one end with no torment and at other end with most exceedingly bad torment ever. Patients are inquired to show where on the line he or she rates the torment on the day of introduction, 2nd week, 6th week and 6th month of follow-up.

## Discussion:

The aim of this study was to evaluate the efficacy of a nutritional supplement containing Glucosamine, type II collagen and vitamin C (orthoboon) on the clinical and structural evolution of tendinopathies. This study was conducted in patients who attended to orthopaedic OPD in Konaseema Institute of Medical Sciences & Research Foundation Amalapuram. The results obtained showed a significant improvement in the four types of tendinopathies included in this study. It should be noted that the four tendons studied (Achilles tendinopathy, patellar tendinopathy, lateral epicondylitis, supra spinatous tendinopathy) have distinctive characteristics, such as the paratenon or type of blood supply, thus meaning that they can be considered to represent anatomical variations of the various tendons present in the body. A visual analog pain scale (VAS) was used to evaluate the improvements observed.

## Advantages of these drugs:

[1] Good patient compliance

[2] No expertise guidance needed.

[3] Non-invasive.

[4] Better relief of pain.

[5] Cost effective.

Age group encountered in the study ranged from 30 to 60 years, with a mean age of 47.2. Peak incidence at 40-50 and 50-60 was noted. The mean age of patients in drug group was 46.65 and in placebo group were 48.85. "P value = 0.2898 which was not significant. Thus age of patients in both groups was comparable. Out of the 80 patients, 58 were males and 22 were females. In drug group 47 were males and 13 were females. In placebo group 11 were males and 9 were females. P value = 0.0429 which is significant. Thus both the group was not comparable, but drug efficacy is tested for further parameters. Out of 80 patients, 62 had their right side involved and 18 had their left side involved. P value = 0.1222 which is not significant. P value for baseline VAS Score is 0.9402 which is statistically not significant. Hence the outcome values before the treatment are comparable. P value for VAS Score at 2nd week is 0.0031 which is statistically highly significant. The decrease in pain at 2nd week is statistically in drug efficacy compared to placebo group. P value II for VAS Score at 2nd month is 0.0001 which is statistically highly significant. Hence the decrease in pain at 2nd month is statistically significant in drug group compared to placebo group P value for VAS Score at 6th month is 0.0001 which is statistically highly significant. Hence the decrease in pain at 6th month is statistically significant in drug group compared to placebo group.

## Conclusion:

Tendinopathy could be a common issue experienced within the orthopedic criteria. These are seen more commonly to affect non-athletes rather than athletes. Much controversy has been there over the pathophysiology and there is not enough scientific evidence so far to favour any particular type of treatment for tendinopathies. Histopathological reports have shown that tendinopathies is not an inflammatory process but a degenerative condition termed Tendinosis'. In this study, glucosamine, collagen II peptide and vitamin C were used for treatment of tendinopathies. At the end of 6 months almost 90% patients relieved completely of pain. The duration of maximum benefit to reach is almost around 24 weeks.
